# Potential links between wood‐inhabiting and soil fungal communities: Evidence from high‐throughput sequencing

**DOI:** 10.1002/mbo3.856

**Published:** 2019-05-27

**Authors:** Witoon Purahong, Katherina A. Pietsch, Helge Bruelheide, Christian Wirth, François Buscot, Tesfaye Wubet

**Affiliations:** ^1^ Department of Soil Ecology UFZ‐Helmholtz Centre for Environmental Research Halle (Saale) Germany; ^2^ Department of Systematic Botany and Functional Biodiversity Leipzig University Leipzig Germany; ^3^ Institute of Biology/Geobotany and Botanical Garden Martin Luther University Halle‐Wittenberg Halle (Saale) Germany; ^4^ German Centre for Integrative Biodiversity Research (iDiv) Halle‐Jena‐Leipzig Leipzig Germany; ^5^ Present address: Department of Community Ecology UFZ‐Helmholtz Centre for Environmental Research Halle (Saale) Germany

**Keywords:** deadwood, fungal dispersal, soil fungi, subtropical forest, wood‐inhabiting fungi

## Abstract

Wood‐inhabiting fungi (WIF) are pivotal to wood decomposition, which in turn strongly influences nutrient dynamics in forest soils. However, their dispersal mechanisms remain unclear. We hypothesized that the majority of WIF are soil‐borne. For this reason, the presented research aimed to quantify the contribution of soil as a source and medium for the dispersal of WIF to deadwood using high‐throughput sequencing. We tested effects of tree species (specifically *Schima superba* and *Pinus massoniana*) on the percentage of WIF shared between soil and deadwood in a Chinese subtropical forest ecosystem. We also assessed the taxonomic and ecological functional group affiliations of the fungal community shared between soil and deadwood. Our results indicate that soil is a major route for WIF colonization as 12%–15% (depending on the tree species) of soil fungi were simultaneously detected in deadwood. We also demonstrate that tree species (*p* < 0.01) significantly shapes the composition of the shared soil and deadwood fungal community. The pH of decomposing wood was shown to significantly correspond (*p* < 0.01) with the shared community of wood‐inhabiting (of both studied tree species) and soil fungi. Furthermore, our data suggest that a wide range of fungal taxonomic (Rozellida, Zygomycota, Ascomycota, and Basidiomycota) and ecological functional groups (saprotrophs, ectomycorrhizal, mycoparasites, and plant pathogens) may use soil as a source and medium for transport to deadwood in subtropical forest ecosystem. While 12%–62% of saprotrophic, ectomycorrhizal, and mycoparasitic WIF may utilize soil to colonize deadwood, only 5% of the detected plant pathogens were detected in both soil and deadwood, implying that these fungi use other dispersal routes. Animal endosymbionts and lichenized WIF were not detected in the soil samples. Future studies should consider assessing the relative contributions of other possible dispersal mechanisms (e.g. wind, water splash, water dispersal, animal dispersal, and mycelial network) in the colonization of deadwood by soil fungi.

## 1 INTRODUCTION

Deadwood represents an important carbon (C) pool in global forest ecosystems, contributing approximately 8%, or 73 petagrams of C, to the total carbon stock (Pan et al., 2011). The decomposition of deadwood is crucial to carbon dynamics and nutrient cycling of forest ecosystems (Fukasawa, 2018; Hoppe et al., 2016; Rajala, Peltoniemi, Pennanen, & Mäkipää, 2012). It is a complex ecological process, influenced by diverse factors, such as climate, substrate quality (e.g. C:N ratio, moisture levels, and lignin content) as well as the abundance, composition, and activity of decomposer communities (Fukasawa, 2018; Liu, Schaefer, Qiao, & Liu, 2013; Purahong, Krüger, Buscot, & Wubet, 2016). Wood‐inhabiting fungi (WIF) are considered to be the most important class of wood decomposers due to the wood decomposition enzymes, for example, oxidoreductases and hydrolases, that they secrete (Purahong, Krüger, et al., 2016).

Advances in metabarcoding approaches using high‐throughput sequencing (HTS) platforms enable detailed analysis of community composition and have been harnessed to reveal a more complete picture of fungal diversity in a wide range of habitats, including soil and deadwood (Goldmann, Schöning, Buscot, & Wubet, 2015; Hiscox et al., 2015; Hoppe et al., 2016; van der Wal, Klein Gunnewiek, Cornelissen, Crowther, & Boer, 2016; van der Wal, Ottosson, & Boer, 2015). Recent studies have demonstrated that diverse taxonomic and ecological functional groups of WIF colonize deadwood (Ottosson et al., 2015; Purahong et al., 2017; Song, Kennedy, Liew, & Schilling, 2016). The functional groups identified include saprotrophs, plant pathogens, endophytes, animal endosymbionts, mycoparasites, mycorrhizae, and lichenized fungi (Ottosson et al., 2015; Purahong et al., 2017; Song et al., 2016). Although several studies have investigated factors related to WIF community assembly (Fukami et al., 2010; Hoppe et al., 2016; Rajala et al., 2012; Song et al., 2016; van der Wal et al., 2015), connections between the WIF community and soil fungi, and dispersal mechanisms responsible for their colonization between these compartments, are still unclear. In forest ecosystems, fungi (in spore or mycelium form) can be transported or dispersed from one place to another by for example (a) wind, (b) splash dispersal, (c) water dispersal, (d) animal dispersal, (e) seed‐borne fungi (i.e. endophytes), or the (f) fungal mycelium network (Dighton & White, 2005; Heaton et al., 2012).

Wind is one of the most common dispersal mechanisms, and may play a significant role in WIF dispersal (Dighton & White, 2005; Jacobsen, Kauserud, Sverdrup‐Thygeson, Bjorbækmo, & Birkemoe, 2017; Peay & Bruns, 2014). Insect dispersal may also play an important role for WIF distribution as several species of saproxylic beetles in temperate forest have been found to be the vectors of many WIF species, including *Fomitopsis pinicola*, *Fomes fomentarius*, *Trichaptum abietinum*, and *Trametes versicolor* (Jacobsen et al., 2017). Nevertheless, we previously found no significant differences in either WIF richness or community composition between insect‐excluded deadwood (of *Schima superba* and *Pinus massoniana*) and control material, and direct insect associated fungi, for example, insect parasites and endosymbionts were seldom detected in the deadwood (Purahong et al., 2017). This finding indicates either that insects are less important for WIF distribution in subtropical forests than in temperate forests or that WIF transported by insect vectors can also reach deadwood via other mechanisms. A large proportion of the deadwood in forest ecosystems is located on the forest floor and will thus come into direct contact with soil at some point of the decomposition process (Song, Vail, Sadowsky, & Schilling, 2015). A previous study in boreal forest found that soil contact is significantly correlated with WIF community composition, implying that WIF in this ecosystem use soil as a medium to colonize deadwood, and that soil contact may influence WIF community assemblage, subsequently affecting the wood decomposition process (Rajala et al., 2012). Hence, fungal transport from soil to deadwood may involve multiple dispersal mechanisms. Following wood colonization, fungal mycelium can grow out into the soil and form a network through cords or rhizomorphs, which, in some species (e.g. *Armillaria* spp.), can cover areas ranging from several square meters to 1,000 ha (Heaton et al., 2012; Mihail & Bruhn, 2005). Such fungal mycelial networks can expand through soil to colonize new food sources, for example, leaf litter and deadwood, located on the surrounding forest floor (Boddy, Hynes, Bebber, & Fricker, 2009). Fungal spores and mycelial fragments are released from deadwood and can be further transported by wind and water to the soil surface and subsurface (Dighton & White, 2005; Nawaz et al., 2016). Thus, soil can be considered both a source and medium for the transport of wood‐inhabiting fungi.

In this study, we had three aims. First, to evaluate the contribution of soil as a source and medium in the colonization of wood‐inhabiting fungi to deadwood using fungal community datasets derived from deadwood (Purahong et al., 2017) and soil samples (Schuldt et al., 2015) collected from a Chinese subtropical forest ecosystem. Second, to test effects of tree species identity on the percentage of WIF shared between soil and deadwood. Third, to assess the taxonomic and ecological functional group affiliations of the fungal communities found in both deadwood and soil. We hypothesized that a significant proportion of wood‐inhabiting fungi from diverse taxonomic and ecological functional groups use soil as both a source and medium for dispersal to deadwood. Tree species identity, as characterized by certain wood physicochemical parameters, was found to be the main factor influencing the taxonomic and functional diversity of soil fungi colonizing deadwood.

## 2 MATERIALS AND METHODS

### 2.1 Fungal community datasets

We integrated published HTS molecular datasets for soil (Schuldt et al., 2015) and deadwood (Purahong et al., 2017) fungal communities derived from 27 comparative study plots (30 m × 30 m) located in the Gutianshan National Nature Reserve (GNNR, 81 km^2^, 29°08′–29°17′N, 118°27′–118°11′E), Zhejiang province, South‐East China, as part of the Biodiversity‐Ecosystem Functioning (BEF‐China) project (Bruelheide et al., 2011). The vegetation here is characterized as moist, mixed subtropical broadleaved forest with successional ages ranging from <20 to ≥80 years (Bruelheide et al., 2011). The study area had a mean annual temperature of 15.1°C, with a minimum of −6.8°C recorded in January and a maximum of 38.1°C measured in July. The elevation within the area ranged from 251 to 903 m a.s.l. and levels of tree and shrub species richness from 25 to 69 species per 900 m^2^ plot. The soil and deadwood samples are suitable for comparison as they were collected from the same plots during the same period (August–September 2012).

Soil fungal communities were sampled from the upper soil layer (0–10 cm) of the 27 forest plots as described by Schuldt et al. (2015). Briefly, eight samples (one of which was collected near the deadwood logs used for WIF community analysis) were collected from each 900 m^2^ plot and pooled to obtain composite samples (Schuldt et al., 2015). WIF were collected from the deadwood (diameter = 10 ± 2 cm and length = 25 ± 1 cm) of two tree species, *S. superba* (family Theaceae) (57 samples) and *P. massoniana* (family Pinaceae) (58 samples), which had been harvested in the vicinity of the study area, placed among the 27 comparative study plots in August 2010 and allowed to decompose for 2 years (Purahong et al., 2017). Following the decomposition period, two 2‐cm thick slices, one from the margin, and one from the center of the sample, were sawed from each deadwood sample, kept frozen at −20°C and transported on dry ice to Germany for further physicochemical and molecular analyses. During the molecular WIF analysis, all of the bark was removed from the deadwood samples before homogenization with liquid nitrogen and a swing mill (Retsch, Haan, Germany).

Microbial DNA was extracted from 1 g portions of the composite freeze‐dried soil samples using a MoBio soil DNA extraction kit (MoBio, Carlsbad, CA) and 100 mg portions of the homogenized wood samples using a ZR Soil Microbe DNA MiniPrep kit (Zymo Research, Irvine, CA) (Purahong et al., 2014). For both sets of material, we used the same primer pairs (ITS1F [5′‐C​T​T​G​G​T​C​A​T​T​T​A​G​A​G​G​A​A​G​T​A​A​‐3′] and ITS4 [5′‐T​C​C​T​C​C​G​C​T​T​A​T​T​G​A​T​A​T​G​C​‐3′]) to amplify the entire fungal internal transcribed spacer (ITS) rRNA region (Gardes & Bruns, 1993; White, Bruns, Lee, & Taylor, 1990). The amplified fragments were then subjected to 454 pyrosequencing and bioinformatics analysis as described in detail by Purahong, Wubet, et al. (2016), Purahong et al. (2017) and Schuldt et al. (2015). We did a unidirectional sequencing of the amplicons from the ITS4 end (reverse primer), thus the fungal ITS2 sequences were used for further analysis. The entire ITS region (Tedersoo et al., 2015) and ITS2 is recommended for metabarcoding (Ihrmark et al., 2012; Tedersoo et al., 2015). Fungal ITS OTU representative sequences were first classified against the dynamic version of the UNITE fungal ITS sequence database (version 6, released on January 15, 2014; Kõljalg et al., 2013). The sequences with fungi only identified were further classified against the full version of the UNITE database to improve their taxonomic annotation. We checked the taxonomic annotation of 123 fungal OTUs used in this study by BLAST search against the current version of UNITE (version: 8.0; 2018‐12‐08) and UNITE species hypotheses (Nilsson et al., 2019) of each OTU is presented in Table S1, Supporting Information. Representative sequences of fungal operational taxonomic units (OTUs) were assigned to ecological functional groups based on sequence similarity (≥90%) using the default parameters of the GAST algorithm (Huse et al., 2008) against the reference dataset (Tedersoo et al., 2014). In addition, FUNGuild was also used to assign the ecological functional groups of WIF (Nguyen et al., 2016). A comparison of the results obtained by these functional group assignment approaches is presented in Table S1, Supporting Information.

### 2.2 Statistical analysis

In this study, we focused on the fungal OTUs (123) detected in both soil and deadwood samples. As the HTS sequence datasets were processed together, the OTUs present in both datasets refer to the same fungi. The data concerning shared communities that were used for statistical analysis are provided in the Supporting Information (Table S1). Three‐dimensional non‐metric multidimensional scaling (3D‐NMDS) ordination based on presence/absence data and the Jaccard dissimilarity measure coupled with the *envfit* function of the vegan package in *R* were used to investigate and visualize correlations among the factors that influence shared soil‐deadwood fungal community composition in *P. massoniana* and *S. superba*. 3D‐NMDS worked better than a 2D‐NMDS for our data, as a result of a lower stress value for the former. We repeated 3D‐NMDS coupled with *envfit* for each tree species to determine the factors that influence shared soil‐deadwood fungal community composition in the respective species (*P. massoniana* and *S. superba*). The effect of tree species on the shared soil‐deadwood fungal composition was analyzed using PERMANOVA based on presence/absence data and the Jaccard dissimilarity measure in the PAST program version 2.17c (Hammer, Harper, & Ryan, 2001). Statistical significance was based on 999 permutations. The HTS dataset of wood‐inhabiting and soil fungi was deposited in the European Bioinformatics Institute database under the study numbers PRJEB8978 and PRJEB8979, respectively (https://www.ebi.ac.uk/ena/data/view/PRJEB8978 and https://www.ebi.ac.uk/ena/data/view/PRJEB8979).

## 3 RESULTS AND DISCUSSION

### 3.1 Soil is an important route of WIF dispersal

**Figure A1:**
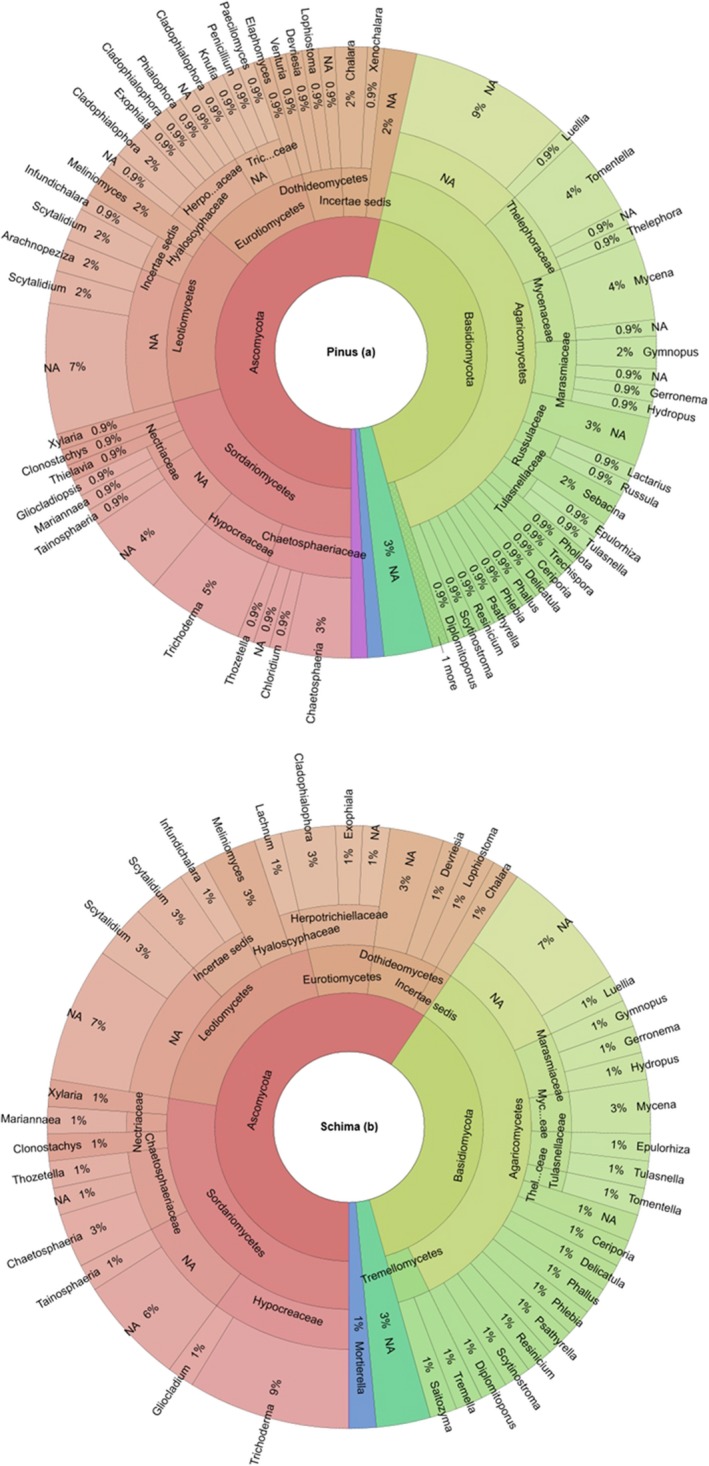
Wood‐inhabiting fungal community composition associated with *Pinus massoniana* (a) and *Schima superba* (b) deadwood calculated using presence‐absence data for the 123 fungal OTUs detected in both deadwood and soil samples (*Pinus massoniana* = 116 OTUs and *Schima superba* = 69 OTUs). Herpo…aceae = Herpotrichiellaceae; Tric…ceae = Trichocomaceae; Myc…eae = Mycenaceae; Thel…ceae = Thelephoraceae

Our results demonstrate that soil is an important route for the colonization of soil fungi to deadwood in Chinese subtropical forest ecosystem. A taxonomically diverse array of fungi with various ecological functional groups was detected in soil and deadwood samples (Table 1; Figure A1). We detected a total of 123 fungal OTUs in both soil and deadwood samples. This finding suggests that at least 12% of the total WIF community (997 detected OTUs, of which 12% and 15% were detected in *S. superba* and *P. massoniana*, respectively) use soil as a source and transport medium to deadwood. This proportion of fungal OTUs shared between soil and deadwood identified in the Chinese subtropical forest is consistent with a previously reported proportion in a temperate forest (~10%), but much lower than the reported proportion in a boreal forest (~50%) (Mäkipää et al., 2017; van der Wal, klein Gunnewiek, & de Boer, 2017). This may be related to differences in biomes and/or the role of deadwood in each biome (Mäkipää et al., 2017; Purahong et al., 2017; van der Wal et al., 2017). Interestingly, despite similarities in fungal community composition in the soil and minimum distance between the deadwood of the two tree species (*S. superba* and *P. massoniana*) in each experimental plot, numbers of fungal OTUs detected in soil and deadwood of the two species differed (Table 1). *P. massoniana* harbored most of the OTUs (94%) shared between soil and wood‐inhabiting fungi; among these shared OTUs, 54 (46.5%) were detected in *P. massoniana* but not *S. superba* wood. In contrast, *S. superba* harbored 69 OTUs (56% of the total shared community), most of which were also detected in *P. massoniana* (62 OTUs, 90%), as only seven (10%) WIF OTUs were specifically associated with this tree species.

**Table 1:** Numbers of total, specific, and shared wood‐inhabiting fungal OTUs (separated according to ecological functional groups) identified in soil and *Pinus massoniana* and *Schima superba* deadwood samples

Functional group	Total number of OTUs detected in soil	Total number of OTUs detected in deadwood	Soil and deadwood shared OTUs	Total number of OTUs detected in soil and deadwood samples		
				*Pinus* specific	*Schima* specific	Shared
Animal endosymbiont	0	1	0	0	0	0
Animal parasite	14	0	0	0	0	0
arbuscular mycorrhiza	51	0	0	0	0	0
Ectomycorrhiza	534	21	13	11	0	2
Endophyte	1	0	0	0	0	0
Lichenized	2	12	0	0	0	0
Mycoparasite	11	17	5	0	2	3
Plant pathogen	73	44	2	1	0	1
Saprotroph	980	652	76	29	2	45
Unknown	539	250	27	13	3	11
Summary	2,205	997	123	54	7	62

Note

The shared OTUs represent fungi that may use soil to colonize the deadwood of the two studied tree species.

Total number of OTUs detected in *P. massoniana* deadwood = 790 OTUs and in *S. superba* deadwood = 583 OTUs.

**Figure 1:**
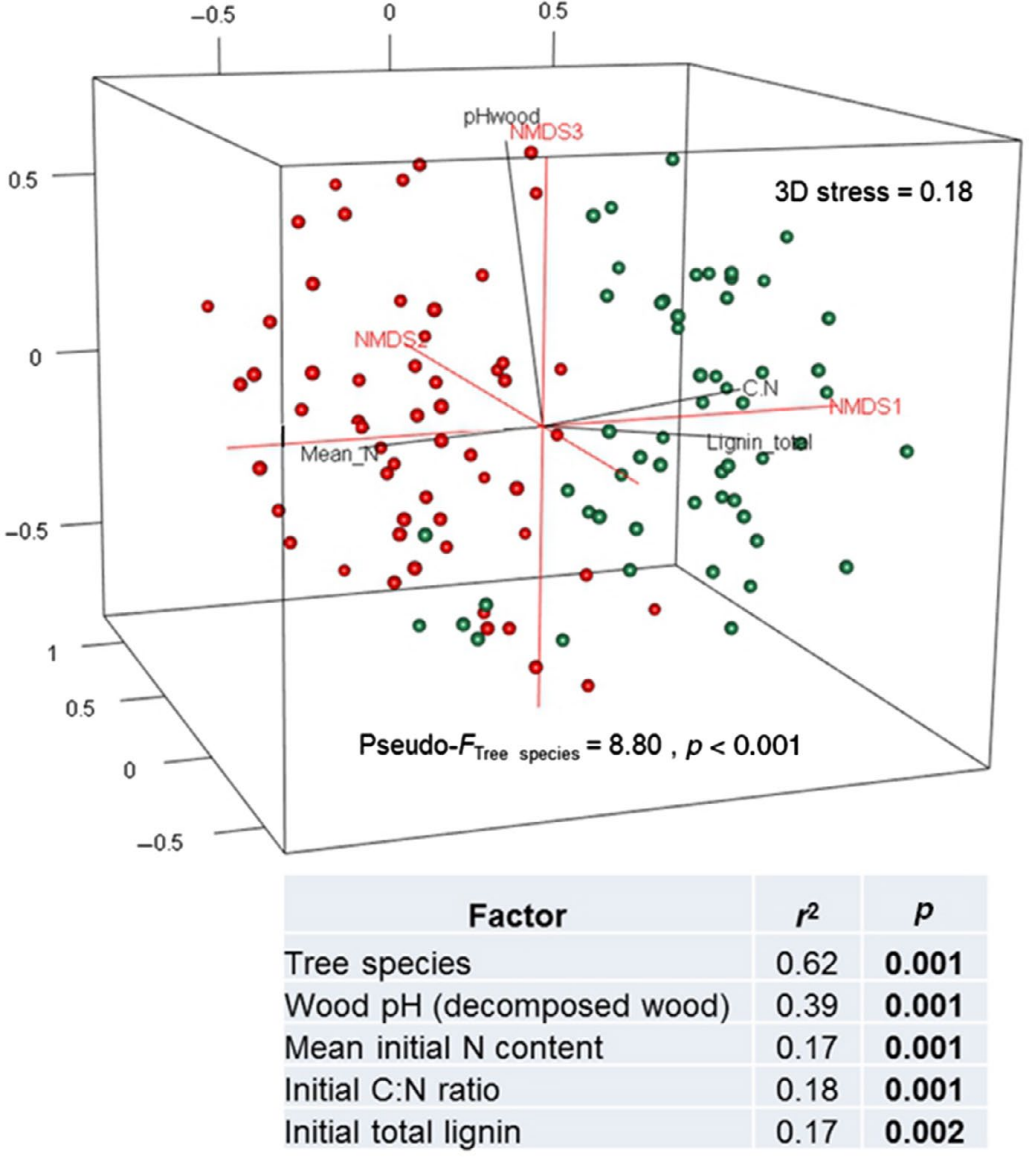
Three‐dimensional non‐metric multidimensional scaling (3D‐NMDS) ordinations of wood‐inhabiting fungal community composition in *Pinus massoniana* (green) and *Schima superba* (red) deadwood (calculated using data for the 123 fungal OTUs detected in both deadwood and soil samples) based on presence/absence data and the Jaccard dissimilarity measure. The NMDS ordination was fitted to factors describing wood physicochemical properties (significant factors *p* < 0.01 are shown as vectors, with statistical values presented in the table). PERMANOVA using presence/absence data and the Jaccard dissimilarity measure was used to test the effect of tree species on wood‐inhabiting fungal community composition (statistical significance is based on 999 permutations)

Furthermore, the two species differed significantly in terms of WIF community transported via the soil (PERMANOVA, *F* = 8.80 (presence/absence data, Jaccard dissimilarity measure), *p* < 0.001, 999 permutations, Figure 1). Spatial distance is one of the most important drivers of fungal community assembly, and this factor could have affected the validity of the experiment (Peršoh, 2015; Purahong, Krüger, et al., 2016; Talbot et al., 2014). However, we minimized the effect of spatial distance by spacing deadwood samples from the two tree species at a set minimum distance in each experimental plot. Thus, our results clearly indicate that tree species identity as defined by wood physicochemical properties including initial N content (highly correlated with initial C content), pH of decomposed wood, initial C:N ratio, and initial total lignin content, strongly influences the WIF community transported from soil to deadwood (Figure 1). The pH of the decomposed wood samples was significantly correlated with the shared soil and deadwood fungal community in both *S. superba* and *P. massoniana* (Table 2). This is consistent with findings that under controlled conditions WIF species can significantly change the pH of colonized deadwood after 2–4 weeks (Humar, Petrič, & Pohleven, 2001). pH changes in decomposing wood can influence subsequent fungal communities as the optimal pH for fungal growth and reproduction rates is species‐specific (Hoppe et al., 2016; Purahong, Krüger, et al., 2016; Yamanaka, 2003).

**Table 2:** Goodness‐of‐fit statistics (*r* ^2^) for factors fitted to the three‐dimensional non‐metric multidimensional scaling (3D‐NMDS) ordinations of wood‐inhabiting fungal community composition in *Pinus massoniana* and *Schima superba* deadwood (calculated using data for 116 and 69 fungal OTUs shared between soil samples and deadwood of *P. massoniana* and *S. superba*, respectively) based on presence/absence data and the Jaccard dissimilarity measure

Factor	*Pinus massoniana*		*Schima superba*	
	*r* ^2^	*p*	*r* ^2^	*p*
Initial wood physiochemical properties				
Mean N	0.02	0.800	0.06	0.325
C: N ratio	0.03	0.676	0.07	0.281
Lignin content	0.01	0.883	0.01	0.955
Decomposing wood properties				
Wood pH	0.32	**0.001**	0.25	**0.002**

Note

Statistical significance is based on 999 permutations.

### 3.2 Relative proportion of the fungal phyla, classes, and families transported from soil to deadwood

Our data suggest that the two dominant fungal phyla in deadwood—Ascomycota and Basidiomycota (Hoppe et al., 2016; Purahong, Wubet, Krüger, & Buscot, 2018; Rajala et al., 2012)—may both be potentially dispersed to deadwood via the soil in Chinese subtropical forest ecosystem. This pattern was consistent in both studied tree species (Ascomycetes and Basidiomycetes accounting for 53%–59% and 36%–42% of the total shared community, respectively) (Figure A1). In addition, Zygomycota and Rozellida fungi may be dispersed via soil. The Ascomycota identified in *S. superba* and *P. massoniana* deadwood mainly belonged to four classes: Sordariomycetes (represented families: Hypocreaceae and Chaetosphaeriaceae); Leotiomycetes (represented family: Hyaloscyphaceae); Eurotiomycetes (represented family: Herpotrichiellaceae); and Dothideomycetes. The Basidiomycota identified in the samples were predominantly from the class Agaricomycetes (represented families: Mycenaceae, Marasmiaceae, and Thelephoraceae). At fine taxonomic resolution, that is, genus and OTU levels, the two tree species differed greatly in terms of WIF community composition (Figure A1). Notably, although the same pool of fungal OTUs was present in the soil of all experimental plots, specific sub‐pools of these OTUs successfully colonized deadwood of each tree species (Figure 1), in accordance with previous findings (Hoppe et al., 2016). We conclude that fungi from the phyla Ascomycota and Basidiomycota potentially use the soil as a source and transport medium to colonize deadwood. We also provide evidence that other fungal phyla which are less frequently detected in deadwood, such as Zygomycota and Rozellida, may use soil as a medium for dispersal to deadwood.

### 3.3 Proportion of WIF functional groups dispersed via the soil medium

Diverse functional groups of fungi are present in deadwood (Ottosson et al., 2015; Purahong et al., 2017), but not all of them are transported to deadwood via soil. We expected to find that free‐living fungal functional groups that can inhabit either soil or detritus spheres (such as saprotrophic fungi) can more readily use soil as a means to colonize deadwood than mycoparasites, endophytes (fungal endophytes and animal endosymbionts), or plant pathogens. This is because mycoparasites, endophytes, and plant pathogens have complex lifestyles, requiring not only fungal propagules, but also suitable hosts, to be present in or on the deadwood following dispersal. However, we found that saprotrophs, ectomycorrhiza, mycoparasites, and plant pathogens are all potentially transported via soil (Table 1). Substantial proportions of saprotrophic, ectomycorrhizal, and mycoparasitic fungi may be dispersed by soil, especially ectomycorrhizal fungi, with the genera *Tomentella*, *Elaphomyces*, *Lactarius*, *Russula*, *Sebacina*, and *Thelephora* accounting for 62% of the total WIF ectomycorrhizal OTUs detected in both soil and deadwood samples (Table 1). A recent study also found that a high proportion of ectomycorrhizal fungi (85%) in boreal forest use soil as a source and medium for transport to deadwood (Mäkipää et al., 2017). However, for Thelephorales (e.g. *Tomentella*), insects may also play a large role in their dispersal (Lilleskov & Bruns, 2005). It should be noted that although the role of ectomycorrhizal fungi in deadwood decomposition remains unclear, there is increasing evidence that certain ectomycorrhizal fungi may be facultative saprotrophs (Lindahl & Tunlid, 2015; Rajala et al., 2012; Rajala, Tuomivirta, Pennanen, & Mäkipää, 2015).

The most commonly detected WIF saprotrophs in our samples included *Resinicium* Otu 00870 (UNITE species hypotheses: *Resinicium friabile* (SH1145397.08FU), the most frequently detected WIF in the deadwood dataset), *Psathyrella* Otu 00072 (UNITE species hypotheses: *Psathyrella candolleana* (SH1233511.08FU)), *Scytinostroma* Otu 01080 (SH1181835.08FU), *Xylaria* Otu 01638 (SH1170105.08FU), and *Phlebia* Otu 02299 (UNITE species hypotheses: *Phlebia tuberculate* (SH1175940.08FU)), all of which were detected in soil sample (Table S1, Supporting Information and Table A1). All of these WIF OTUs were detected less frequently in soil samples than in deadwood samples (Table A1). This implies that different WIF propagules from varying ecological functional groups use soil as a source and transport medium for colonizing deadwood and, once the propagules have reached the deadwood, colonization success depends on the competence of the species to coexist with other fungal taxa (which also determines the ecological significance of the species in deadwood decomposition). A summary of all the 123 WIF OTUs detected in both soil and deadwood samples is presented in Table A1.

Plant pathogenic WIF were the second most diverse functional group, in terms of OTUs, detected in the deadwood dataset (Purahong et al., 2017). However, our results indicate that only two of these OTUs (*Devriesia* Otu 01032 (UNITE species hypotheses: *Devriesia* sp. [SH1222449.08FU]) and *Venturia* Otu 01081(UNITE species hypotheses: *Venturia* [SH1222290.08FU]) are potentially transported to deadwood via soil. These findings indicate that plant pathogenic WIF may be largely transported by other means, for example via air dispersal, or that these fungi initially infect living plants and can subsequently switch to saprotrophic growth. If so, the deadwood may also serve as inoculum (Maharachchikumbura, Hyde, Groenewald, Xu, & Crous, 2014). WIF representing other ecological functional groups, including animal endosymbionts and lichenized fungi, were not detected in the soil samples, indicating that WIF of such functional groups are transported via routes other than soil. Animal endosymbiont WIF could be transported through insect vectors (Dighton & White, 2005). The dispersal of lichenized WIF is much more complex, as not only fungi, but also a compatible algal or cyanobacterial partner, must either be transported to or be presented on the colonized deadwood (Dal grande, Widmer, Wagner, & Scheidegger, 2012).

### 3.4 Links between wood‐inhabiting and soil fungal communities

There are four hypotheses for a shared occurrence of fungal OTUs between the deadwood and the soil (Mäkipää et al., 2017 and this study): (a) the fungal OTUs migrated from the soil to the wood, (b) the fungal OTUs migrated from the wood to the soil, (c) the fungal OTU migrated from somewhere else to both the wood and the soil, and (d) the fungal OTUs are ubiquitous, generalist organisms that were present in both the soil and the wood. In our study we were not able to quantify the relative important of each hypothesis but we can assess the overall contribution of soil as source and medium for transport of wood‐inhabiting fungi to deadwood. We expected the number of fungal OTUs migrating from the initial deadwood to the soil to be low, as we did not detect any of the fungal endophytes in the fungal community shared between soil and deadwood. Fungal OTUs that use soil as source and/or medium to colonize deadwood could follow any of the four migratory patterns, because they must be transported to, survive in, and/or colonize the soil. As we removed the bark from deadwood samples before homogenization and DNA extraction, fungi attached to the bark that had not penetrated the inner tissue of the deadwood (i.e. soil‐ or wind‐dispersed fungal spores that had attached to the bark) should have been excluded from our analysis. Mäkipää et al. (2017) studied on soil‐ and deadwood‐inhabiting fungal communities in boreal forests and found that these communities interact along the decay gradient of Norway spruce logs, and a relatively high proportion of the total fungal community is present in both soil and deadwood. Mäkipää et al. (2017) also found that WIF locally influence the soil‐inhabiting fungal communities at all decay stages because certain WIF only occur in the soil under specific decaying logs, but it is impossible to determine how many soil fungal OTUs colonized the deadwood due to the study's experimental set‐up. To enable this, a long‐term, time‐series analysis (from initial decay to late decay stages) of the fungal OTUs present in both soil and deadwood would be required.

### 3.5 Potential biases of the fungal datasets

In this study the sequence data were generated with the pyrosequencing sequencing technology (no longer available). However, we achieved substantial numbers of sequence reads per sample to reasonably infer the fungal diversity in soil and deadwood. The sequencing depths of 10,000 and 3,077 sequences per sample were used for the soil and deadwood samples, respectively (Purahong et al., 2017; Purahong, Wubet, et al., 2016; Schuldt et al., 2015). The primer used (ITS1F and ITS4) represent genetic markers known to carry possible bias toward amplification of basidiomycetes and ascomycetes, respectively (Bellemain et al., 2010). We thus, carefully interpreted the results from this study as with the current primer set we may not amplify the total taxa of Zygomycota and other fungal phyla.

## 4 CONCLUSION AND RESEARCH PERSPECTIVE

Different fungal taxonomic (including Rozellida, Zygomycota, Ascomycota, and Basidiomycota) and functional groups use soil as a source and transport medium to colonize deadwood. Tree species identity, characterized by wood physicochemical parameters including C, N, and total lignin contents, as well as the C:N ratio of undecomposed wood and pH of the decomposed wood, was found to significantly impact the WIF community that colonized deadwood via soil. Substantial proportions of saprotrophic, ectomycorrhizal, and mycoparasitic fungi may be transported via soil. However, plant pathogens, animal endosymbionts, and lichenized fungi seem to reach deadwood via other routes. Even though our results indicate that soil is a major route for deadwood fungal colonization (accounting for 12%–15% of the total WIF fungal community present in both soil and deadwood) we suggest that future studies should consider and evaluate other possible dispersal mechanisms for the colonization of deadwood by soil fungi (e.g. wind, water splash, run‐off, animals, and mycelial network) to gauge their respective contributions to deadwood colonization and decomposition.

## CONFLICT OF INTERESTS

None declared.

## AUTHOR CONTRIBUTIONS

T.W. and W.P. conceived the study, W.P. and T.W. analyzed the data and wrote the manuscript, K.P., C.W., T.W., H.B., and F.B. setup the field experiments. F.B., H.B., C.W., and K.P. contributed data and edited the manuscript.

## ETHICS STATEMENT

None required.

## DATA ACCESSIBILITY

The high‐throughput sequencing dataset of wood‐inhabiting and soil fungi were deposited in the European Bioinformatics Institute database under the study numbers PRJEB8978 and PRJEB8979, respectively (https://www.ebi.ac.uk/ena/data/view/PRJEB8978 and https://www.ebi.ac.uk/ena/data/view/PRJEB8979).

## APPENDIX 1

**Table A1:** Total sequences (sum of the number of all detected sequences; total sequence abundance) of 123 WIF OTUs in soil and deadwood samples

Fungal OTU	Function	Total sequences in soil	Total sequences in deadwood
*Resinicium* Otu 00870	Saprotroph	Below 100	86,475
*Scytinostroma* Otu 01080	Saprotroph	Below 100	14,854
*Scytalidium* Otu 01766	Unknown	Below 100	11,716
*Psathyrella* Otu 00072	Saprotroph	636	7,704
*Phlebia* Otu 02299	Saprotroph	Below 100	6,407
*Xylaria* Otu 01638	Saprotroph	Below 100	6,103
Agaricales Otu 00019	Saprotroph	1511	3,144
*Phallus* Otu 01000	Saprotroph	Below 100	2,907
*Gerronema* Otu 00430	Saprotroph	Below 100	2,751
*Mariannaea* Otu 00714	Saprotroph	Below 100	2,617
*Infundichalara* Otu 00820	Saprotroph	Below 100	1,625
*Gymnopus* Otu 00349	Saprotroph	111	1,488
Hysterangiales Otu 00521	EcM	Below 100	1,395
*Mycena* Otu 00403	Saprotroph	Below 100	1,343
Herpotrichiellaceae Otu 01865	Saprotroph	Below 100	1,300
Sordariales Otu 01838	Saprotroph	Below 100	1,286
*Luellia* Otu 00798	Unknown	Below 100	1,276
*Pholiota* Otu 01444	Saprotroph	Below 100	1,179
Helotiales Otu 01418	Unknown	Below 100	910
*Scytalidium* Otu 01387	Saprotroph	Below 100	904
*Phialophora* Otu 01634	Saprotroph	Below 100	636
*Chaetosphaeria* Otu 01924	Saprotroph	Below 100	621
*Epulorhiza* Otu 01242	Saprotroph	Below 100	599
*Delicatula* Otu 01073	Saprotroph	Below 100	529
*Trichoderma* Otu 00671	Saprotroph	Below 100	442
*Mycena* Otu 00060	Saprotroph	723	416
Chaetosphaeriaceae Otu 00090	Saprotroph	547	402
*Chalara* Otu 00894	Saprotroph	Below 100	392
*Tulasnella* Otu 01580	Saprotroph	Below 100	364
*Hydropus* Otu 00436	Saprotroph	Below 100	322
*Scytalidium* Otu 00316	Saprotroph	127	280
*Cladophialophora* Otu 02309	Saprotroph	Below 100	277
*Tainosphaeria* Otu 01907	Unknown	Below 100	213
Auriculariales Otu 00652	Saprotroph	Below 100	200
Agaricomycetes Otu 01452	Unknown	Below 100	198
*Venturia* Otu 01081	Plant pathogen	Below 100	186
*Trichoderma* Otu 00162	Mycoparasite	319	178
*Lophiostoma* Otu 01281	Saprotroph	Below 100	175
*Meliniomyces* Otu 00801	Saprotroph	Below 100	146
*Arachnopeziza* Otu 01681	Saprotroph	Below 100	134
Rozellida Otu 00154	Unknown	342	132
Rozellida Otu 00270	Unknown	157	130
Sebacinales Otu 00076	Unknown	627	108
*Xenochalara* Otu 01231	Saprotroph	Below 100	101
*Chaetosphaeria* Otu 00392	Saprotroph	Below 100	93
*Mycena* Otu 00187	Saprotroph	263	81
Leotiomycetes Otu 01420	Unknown	Below 100	81
Trechisporales Otu 00031	Saprotroph	1,194	77
Pleosporales Otu 02316	Unknown	Below 100	76
*Trechispora* Otu 00161	Saprotroph	319	64
*Tomentella* Otu 00191	EcM	256	64
Thelephoraceae Otu 00221	EcM	200	62
*Diplomitoporus* Otu 00615	Saprotroph	Below 100	62
*Chaetosphaeria* Otu 00717	Saprotroph	Below 100	59
*Tomentella* Otu 00002	EcM	5,501	56
Helotiales Otu 00738	Unknown	Below 100	55
Sordariomycetes Otu 01975	Unknown	Below 100	55
Helotiales Otu 00300	Unknown	136	53
*Chalara* Otu 00582	Saprotroph	Below 100	49
*Scytalidium* Otu 01896	Saprotroph	Below 100	44
*Tomentella* Otu 00130	EcM	398	41
*Knufia* Otu 01013	Saprotroph	Below 100	36
*Thozetella* Otu 01415	Saprotroph	Below 100	36
*Cladophialophora* Otu 01423	Saprotroph	Below 100	36
Agaricales Otu 00674	Saprotroph	Below 100	30
*Mycena* Otu 00781	Saprotroph	Below 100	28
Rozellida Otu 00732	Unknown	Below 100	27
*Lactarius* Otu 00780	EcM	Below 100	26
Sordariomycetes Otu 02315	Saprotroph	Below 100	25
*Saitozyma* Otu 00001	Saprotroph	9,348	24
*Gymnopus* Otu 01500	Saprotroph	Below 100	24
*Gliocladium* Otu 01778	Mycoparasite	Below 100	23
Trechisporales Otu 00079	Saprotroph	613	21
Rozellida Otu 00669	Unknown	Below 100	21
*Bionectria* Otu 00932	Saprotroph	Below 100	19
*Lachnum* Otu 01209	Saprotroph	Below 100	18
*Cladophialophora* Otu 01531	Saprotroph	Below 100	18
Fungal Otu 02188	Unknown	Below 100	18
*Trichoderma* Otu 00024	Saprotroph	1,400	16
Helotiales Otu 00119	Unknown	421	15
*Trichoderma* Otu 01292	Mycoparasite	Below 100	15
*Devriesia* Otu 01032	Plant pathogen	Below 100	14
*Exophiala* Otu 01460	Saprotroph	Below 100	14
*Sebacina* Otu 00116	EcM	424	13
*Tremella* Otu 01667	Mycoparasite	Below 100	13
Helotiales Otu 00184	Unknown	267	12
Marasmiaceae Otu 00487	Saprotroph	Below 100	12
Helotiales Otu 00110	Unknown	445	11
*Penicillium* Otu 00157	Saprotroph	336	10
Sebacinales Otu 00287	Saprotroph	144	10
*Exophiala* Otu 00953	Saprotroph	Below 100	10
*Mortierella* Otu 00011	Saprotroph	1982	8
Capnodiales Otu 00707	Unknown	Below 100	8
*Mycena* Otu 01577	Saprotroph	Below 100	8
*Tomentella* Otu 01978	EcM	Below 100	8
Helotiales Otu 00040	Unknown	982	7
*Sebacina* Otu 00047	EcM	855	7
Xylariales Otu 00505	Unknown	Below 100	7
*Meliniomyces* Otu 00964	Saprotroph	Below 100	7
*Paecilomyces* Otu 00210	Saprotroph	223	6
*Ceriporia* Otu 00296	Saprotroph	140	6
*Thelephora* Otu 00786	EcM	Below 100	6
*Chloridium* Otu 01114	Saprotroph	Below 100	6
Ascomycota Otu 01120	Unknown	Below 100	6
Auriculariales Otu 01671	Saprotroph	Below 100	6
Agaricales Otu 02176	Saprotroph	Below 100	6
*Arachnopeziza* Otu 02195	Unknown	Below 100	6
*Elaphomyces* Otu 00010	EcM	2,354	5
*Tomentella* Otu 00085	EcM	584	5
Helotiales Otu 00093	Unknown	535	5
Sordariales Otu 00397	Saprotroph	Below 100	5
*Chaetosphaeria* Otu 01118	Saprotroph	Below 100	5
*Thielavia* Otu 01550	Saprotroph	Below 100	5
Sebacinales Otu 00069	Saprotroph	672	4
*Russula* Otu 00104	EcM	490	4
Hyaloscyphaceae Otu 00159	Saprotroph	326	4
Sebacinales Otu 00416	Unknown	Below 100	4
*Cladophialophora* Otu 00461	Saprotroph	Below 100	4
Mycenaceae Otu 00608	Saprotroph	Below 100	4
Ascomycota Otu 01062	Unknown	Below 100	4
*Trichoderma* Otu 01822	Saprotroph	Below 100	4
*Gliocladiopsis* Otu 02201	Saprotroph	Below 100	4
*Trichoderma* Otu 02279	Mycoparasite	Below 100	4
